# Predictors of Response to Imatinib Therapy and Long‐Term Outcomes in Paediatric and Adolescent Patients With Chronic Myeloid Leukaemia

**DOI:** 10.1002/cnr2.70430

**Published:** 2026-01-06

**Authors:** Souvik Saha, Sanjeev Yadav, Manish Kumar Singh, Khaliqur Rahman, Dinesh Chandra, Ruchi Gupta, Rajesh Kashyap

**Affiliations:** ^1^ Department of Hematology Sanjay Gandhi Postgraduate Institute of Medical Sciences Lucknow India

**Keywords:** chronic myeloid leukaemia, imatinib, India, paediatric

## Abstract

**Background:**

Chronic myeloid leukaemia (CML) is an infrequent myeloproliferative neoplasm in the paediatric population as compared to adults. Despite vast progress in understanding the disease biology and therapy of CML‐chronic phase (CML CP), the applicability of risk scoring systems in practice and prognostic factors in children remain grey areas. Hence, we tried to analyse disease characteristics, molecular response to frontline imatinib therapy and its clinical predictors along with long‐term outcomes in our population.

**Materials and Methods:**

In this study, we retrospectively analysed 104 paediatric patients aged ≤ 18 years, diagnosed with CML CP, treated at our centre between 2007 and 2024. Their baseline demographic profile, clinical characteristics, haematological parameters and molecular transcripts as diagnosed by RT‐PCR were recorded. The follow‐up response assessments, including haematological response, molecular response and long‐term survival, were collected from medical records. Risk scores were computed and correlated with clinical outcomes.

**Results:**

Our study included 104 paediatric patients presenting with CML CP. Splenomegaly was a universal feature with a median size of 10 cm below costal margin. The majority of patients belonged to the low‐risk group according to Sokal, EUTOS and ELTS systems. Imatinib was initiated in all of them and was followed up for a median duration of 72 months. Overall, 95.2% patients achieved complete hematologic response (CHR) at 3 months. A total of 42.5% (43/101) patients achieved MMR at 12 months and 54.4% (55/101) patients achieved MMR at 18 months. Sokal scoring system and EMR at 3 months were significantly correlated with the achievement of MMR at 12 months. A total of 11 patients progressed to blast crisis and 10 patients expired in due course of the disease. The 2‐ and 10‐year PFS was 96% and 82%, respectively. CHR at 3 months was significantly predictive of PFS. None of the scoring systems had predictive value for PFS.

**Conclusion:**

This is one of the largest reported data on Indian paediatric CML CP patients with long‐term outcomes. The dynamics of adult CML, including risk scores, do not perfectly fit the scenario of paediatric patients. Hence, further studies and newer strategies are required for optimal management of paediatric CML patients.

## 1 Introduction

Chronic myeloid leukaemia (CML) is a myeloproliferative disease associated with the presence of Philadelphia (Ph) chromosome which results from a reciprocal chromosomal translocation, t(9; 22) (q34; q11), which leads to the formation of constitutively expressing BCR::ABL tyrosine kinase fusion protein [1]. CML in the paediatric population is rare. According to SEER data, CML accounts for only 2% of all paediatric leukaemias below 15 years [2]. There is a scarcity of data on the incidence of paediatric CML in India. Children and adolescents have a more aggressive presentation when compared to adults. A greater proportion of children and adolescents are diagnosed in the advanced phase (accelerated and blast phase), but the proportions have varied in different studies. They also have a relatively larger spleen and higher total leukocyte count (TLC); however, its impact on long‐term prognosis is not well defined [3, 4, 5].

Imatinib, the first tyrosine kinase inhibitor (TKI) developed, has established itself as the first‐line of therapy for paediatric and adolescent patients with CML CP [6, 7, 8, 9]. Post‐imatinib, newer second‐generation TKIs like nilotinib, dasatinib and bosutinib and third‐generation TKI ponatinib has been approved. Studies have reported good responses to imatinib in paediatric CML with 63% patients achieving complete cytogenetic response (CCyR) at 12 months and 59% patients achieving major molecular response (MMR) at 18 months according to a large study in 2018 [10]. There are very few studies from India and other Asian countries, like Japan, relating to paediatric CML patients and their long‐term outcomes [11, 12]. Hence the present study was carried out:

- To review the clinical presentation at diagnosis.
- To assess the response to imatinib therapy in this population group (< 18 years).
- To compare response rates and survival outcomes to imatinib between age groups of < 14 years versus 14–18 years.
- To validate predictive risk scores used in adult CML for response and survival.
- To assess long‐term survival.

## 2 Materials and Methods

### 2.1 Study Population

In this retrospective observational study from Northern India, 104 newly diagnosed patients of CML CP below the age of 18 years, from 2007 to 2024, were analysed. The clinical and laboratory data were retrieved from their medical records. The clinical symptoms, spleen size, peripheral blood counts and quantitative BCR::ABL levels at initial diagnosis and follow‐up at 3, 6, 12 and 18 months and subsequently every 6 months were recorded. The following patients were excluded:

- Age > 18 years.
- Patients with de novo blast crisis.
- Previously treated patients.

### 2.2 Treatment and Monitoring

Imatinib was administered at a standard dose of 260 mg/m^2^, rounded to the nearest 100 mg and absolute maximum dose of 400 mg. The dose was increased to 340 mg/m^2^ in patients failing to achieve the response milestones, with an absolute maximum dose of 600 mg. They were followed up at monthly intervals for the first 3 months followed by once every 3 months. The spleen size and complete blood counts were monitored at each visit. The molecular response was measured by quantitative RT‐PCR for the BCR::ABL/ABL copies using the IS scale at 3, 6, 12 and 18 months interval from initiation of treatment and every 6 months thereafter. All measurements were performed in duplicate and reported as the mean. Complete hematologic response (CHR) was defined as white blood cell count of less than 10 × 10^9^/L, platelet count of less than 450 × 10^9^/L, no immature cells in the peripheral blood and no palpable splenomegaly. The major molecular response was defined as BCR::ABL1 (IS) ≤ 0.1 [1].

### 2.3 Statistical Analysis

Descriptive statistics of the continuous variable were presented using mean ± standard deviation/median while the categorical variable in frequency (%). To compare the means between two groups, an independent samples *t*‐test was used while for proportions between two groups, Fisher exact test was used. Cox regression model was used to compare survival based on different risk factors. Patients who did not reach the time point of 12‐month assessment were censored at their last follow up visit. *p*‐value < 0.05 was considered statistically significant. Statistical analysis was performed using SPSS‐25, IBM, Chicago, USA.

## 3 Results

A total of 104 paediatric and adolescent patients diagnosed with CML CP were included in the study. The median and mean ages of the study population were 14 and 13.5 years, respectively (range: 4–18 years). Fifty‐five (44.7%) patients were below or equal to 14 years. Males constituted 61.5% of the cases and the remaining 38.5% were females (Table 1).

**TABLE 1:** Baseline clinical characteristics and comparison of the clinical features according to age group.

Parameters	Total (*N* = 104)	Group 1, age ≤ 14 years (*n* = 55)	Group 2, age > 14 years (*n* = 49)	*p*
Age (years)				
Mean	13.5 ± 3.9	10.6 ± 3.1	16.7 ± 1.1	
Median	14	11	17	
Range	4–18	4–14	15–18	
Sex				
Male, *N* (%)	64 (61.5)	35 (63.6)	29 (59.2)	0.6
Female, *N* (%)	40 (38.5)	20 (36.4)	20 (40.8)	
Asthenia, *N* (%)	50 (48.1)	26 (52)	24 (48)	1
Weight loss, *N* (%)	29 (27.9)	13 (44.8)	16 (55.2)	0.3
Fever *N* (%)	36 (34.6)	25 (69.4)	11 (30.6)	0.02
Abdominal fullness, *N* (%)	78 (75)	42 (53.8)	36 (46.2)	0.8
Spleen size (cm)				
Mean	10.6 ± 3.7	10 ± 3.6	11.2 ± 3.6	0.8
Median	10	10	12	

The predominant symptoms observed in our study population were abdominal fullness (75%), asthenia (28.1%), fever (34.6%), and weight loss (27.9%). On physical examination, splenomegaly was the predominant finding and was present in 100% of patients. The median spleen size was 10 cm below the costal margin (Table 1).

The median Hb level was 8.6 g/dL, platelet count was 378 × 10^9^/L and TLC was 226 000/mm^3^. Fifty‐nine patients (56.7%) had transcript e14a2, 38 patients (36.5%) had transcript e13a2 and 7 patients (6.7%) had both transcripts. The patients were risk stratified according to the Sokal, EUTOS and ELTS scoring systems. According to the Sokal scoring system, 69.2% of the patients were categorised as low risk, 24.1% as intermediate risk and 6.7% as high risk. EUTOS scoring system categorised 65.4% of patients as low risk and 34.6% as high risk. ELTS scoring classified 52.9% of patients as low risk, 37.5% of patients as intermediate risk and 9.6% of patients as high risk (Table 2).

**TABLE 2:** Baseline laboratory features and risk stratification according to age group.

Parameters	Total (*n* = 104)	Group 1, age ≤ 14 years (*n* = 55)	Group 2, age > 14 years (*n* = 49)	*p*
Haemoglobin level (g/dL)				
Mean (range)	8.9 (4.4–17.8)	8.3 (5.8–16.3)	9.7 (4.4–17.8)	0.01
Median	8.6	8.0	9.4	
Platelet count (× 10^9^/L)				
Mean (range)	378 (84–1611)	396 (93–1611)	358 (84–999)	0.3
Median	317	317	318	
Total leukocyte count (× 10^9^/L)				
Mean (range)	226 (6–620)	248 (11–474)	202 (6–620)	0.08
Median	217	234	168	
Patients with < 1 lakh, *N* (%)	20 (19.2)	7 (12.7)	13 (26.5)	
Patients with ≥ 1 lakh, *N* (%)	84 (80.8)	48 (87.3)	36 (73.5)	
Transcript type				
E14a2, *N* (%)	59 (56.7)	31 (56.4)	28 (57.2)	0.9
E13a2, *N* (%)	38 (36.5)	20 (36.4)	18 (36.7)	
Both, *N* (%)	7 (6.7)	4 (7.2)	3 (6.1)	
Splenomegaly (cm)				
≤ 8 cm BCM, *N* (%)	24 (23.1)	16 (29.1)	8 (16.4)	0.1
> 8 cm BCM, *N* (%)	80 (76.9)	39 (70.9)	41 (83.6)	
Sokal index				
Low risk (< 0.8), *N* (%)	72 (69.2)	42 (76.3)	30 (61.2)	0.2
Intermediate (0.8–1.2), *N* (%)	25 (24.1)	10 (18.2)	15 (30.6)	
High risk (> 1.2), *N* (%)	7 (6.7)	3 (5.5)	4 (8.2)	
EUTOS scores				
Low risk (< 87), *N* (%)	68 (65.4)	37 (67.2)	31 (63.2)	0.6
High risk (> 87), *N* (%)	36 (34.6)	18 (32.8)	18 (36.8)	
ELTS score				
Low risk (≤ 1.5680), *N* (%)	55 (52.9)	34 (61.8)	21 (42.8)	0.1
Intermediate risk (1.5681–2.2185), *N* (%)	39 (37.5)	17 (31)	22 (44.9)	
High risk (> 2.2185), *N* (%)	10 (9.6)	4 (7.2)	6 (12.3)	

Of the 104 patients, 101 patients reached the defined time point of 12 months for response assessment. The median starting imatinib dose was 300 mg (range 100–400 mg). Table 3 shows the starting dose of the entire cohort. Sixty‐one patients required dose escalation due to non‐achievement of response milestones. The median duration from diagnosis to start of imatinib therapy was 23 days. The entire cohort was followed up for a median duration of 72 months. Overall, 95.2% (99/104) patients achieved CHR at 3 months and 54.8% (57/104) patients achieved early molecular response (EMR). A total of 41.3% (43/104) of the patients achieved MMR at 12 months; when restricted to patients who reached 12 months of therapy, MMR was 42.5% (43/101). 52.9% (55/104) patients achieved MMR at 18 months; when restricted to patients with available 18 months assessment, MMR was 54.4% (55/101). Among the 58 patients who did not achieve MMR at 12 months, tyrosine kinase domain (TKD) mutation status was available for 28 patients. E255K mutation was the commonest (9/28) followed by T315I mutation (7/28). The other mutations observed were G250E, M351T and Y253F. No TKD mutations were detected in 8/28 patients. Eleven (10.5%) patients progressed to blast crisis (six to lymphoid and five to myeloid blast crisis). Ten patients expired in due course of disease, all due to progression to blast crisis. Table 4 shows the time to progression and death in different patients from initiation of imatinib. The median time to progression was 55 months (range 6–124 months) and median time to death was 57 months (range 7–136 months).

**TABLE 3:** Starting dose of imatinib in the study population.

Starting dose (mg)	No. of patients (%)
100	20
200	30
300	24
400	30

**TABLE 4:** Time to progression to blast crisis and death from initiation of imatinib in different patients.

Patient	Time of progression to BC from initiation of imatinib (months)	Time to death from initiation of imatinib (month)
1	75	75
2	56	60
3	54	54
4	124	136
5	67	67
6	24	24
7	6	7
8	95	96
9	18	18
10	11	11
11	3	—

There was no correlation of any variable with CHR at 3 months or EMR. Sokal scoring system was significantly associated with MMR at 12 months (*p* = 0.02 by chi‐square test). EUTOS and ELTS scoring was not predictive of MMR at 12 months. No variable retained significance with MMR at 18 months (Table 5). EMR was an important predictive factor for achievement of MMR. There was significant correlation of EMR at 3 months predicting MMR at 12 and 18 months (*p* < 0.01 by Fisher exact test).

**TABLE 5:** Outcomes according to risk stratification scores.

Events	Low risk, *N* = 72 (69.2%)	Intermediate risk, *N* = 25 (24.1%)	High risk, *N* = 7 (6.7%)	*p*
Sokal score				
CHR at 3 months	68 (94.4%)	24 (96%)	7 (100%)	0.7
MMR at 12 months	35 (48.6%)	5 (20%)	3 (42.8%)	0.02
MMR at 18 months	40 (55.5%)	10 (40%)	5 (71.4%)	0.1

Events	Low risk, *N* = 68 (65.4%)	Not applicable	High risk, *N* = 36 (34.6%)	*p*
EUTOS score				
CHR at 3 months	65 (95.5%)		34 (94.4%)	1
MMR at 12 months	32 (47%)		11 (30.5%)	0.1
MMR at 18 months	36 (52.9%)		19 (52.7%)	1

Events	Low risk, *N* = 55	Intermediate risk, *N* = 39	High risk, *N* = 10	*p*
ELTS score				
CHR at 3 months	53 (96.4%)	37 (94.9%)	9 (90%)	0.6
MMR at 12 months	26 (47.2%)	15 (38.4%)	2 (20%)	0.2
MMR at 18 months	30 (54.5%)	19 (48.7%)	6 (60%)	0.7

**FIGURE 1:**
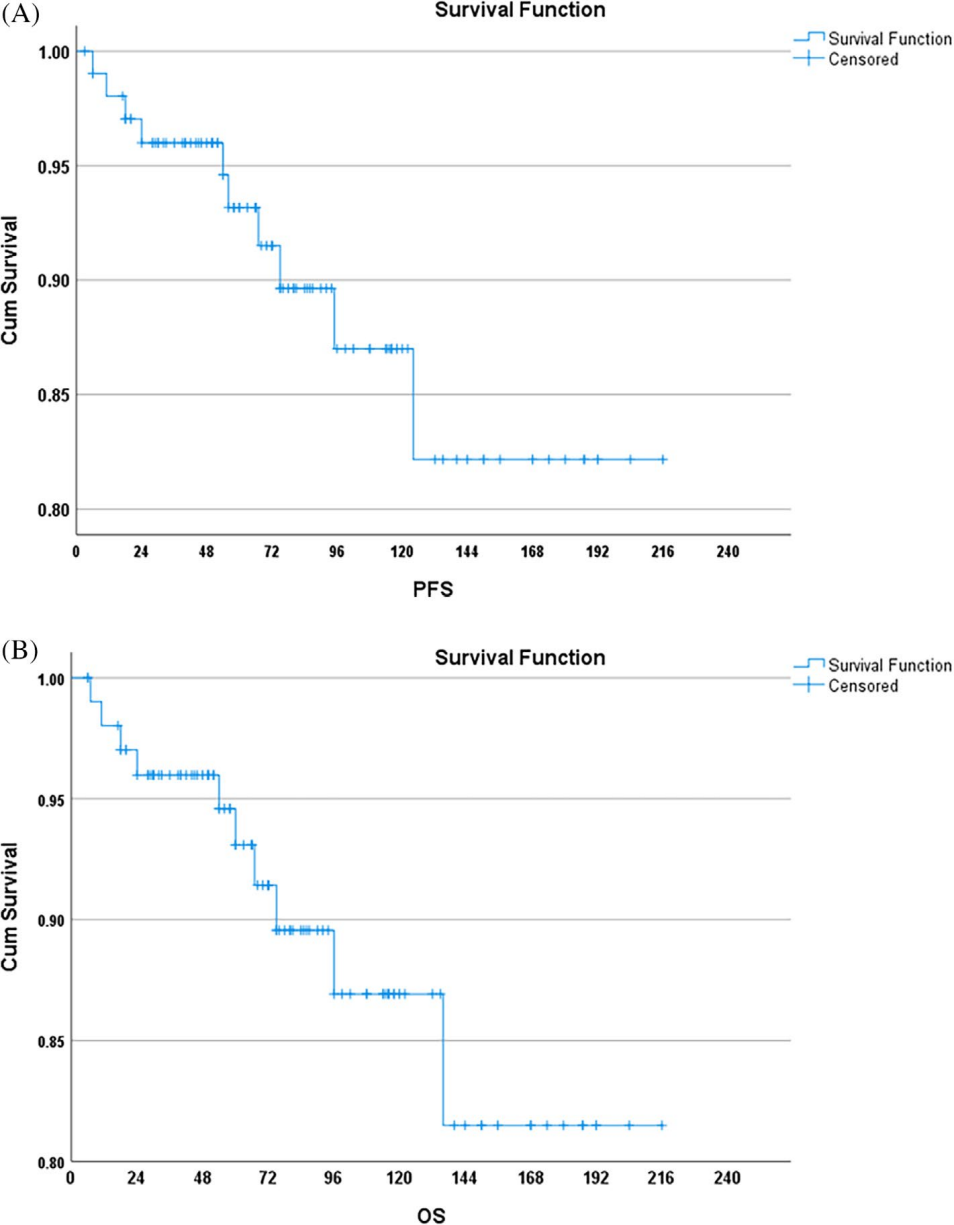
(A) PFS and (B) OS of the entire cohort. Curves begin with the full cohort (*N* = 104) at time zero. Deaths were treated as events at the time of occurrence.

**FIGURE 2:**
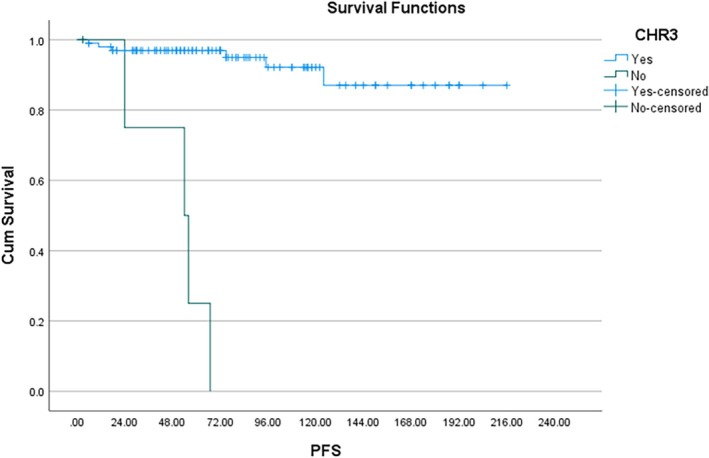
PFS according to CHR at 3 months (*p* < 0.05).

**FIGURE 3:**
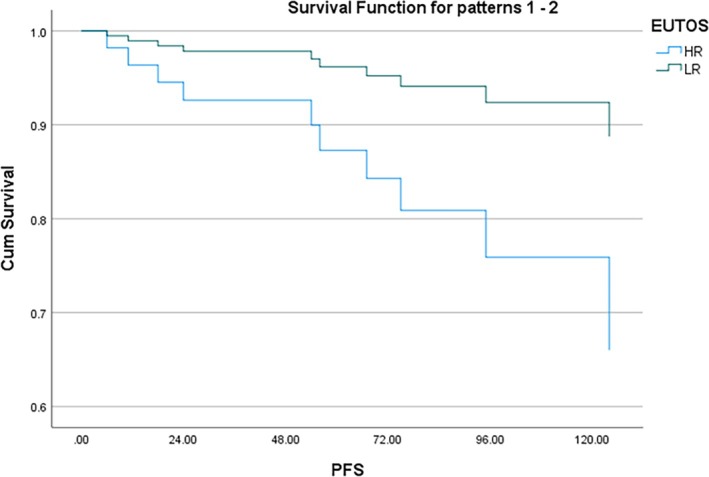
PFS according to EUTOS score (*p* = 0.059).

**FIGURE 4:**
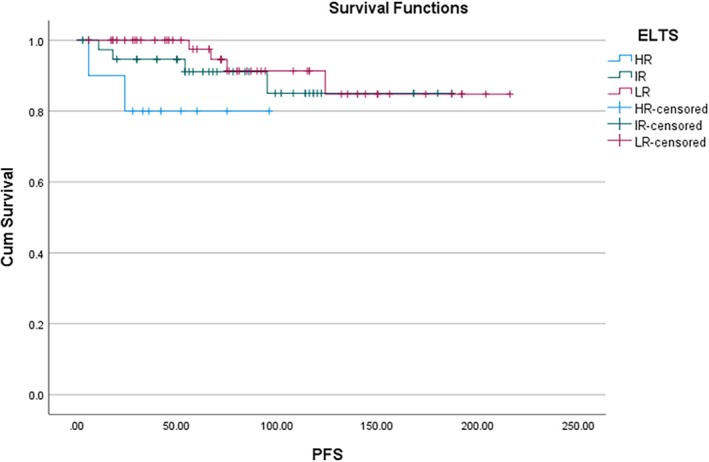
PFS according to ELTS score (*p* = 0.4).

In the Kaplan–Meier analysis, all 104 patients entered the risk set at time zero. Deaths were counted as events at their respective times, resulting in the observed stepwise decrease in survival probability. Thus, the label ‘*N* = 104’ reflects the initial cohort size, not the number of patients alive at each timepoint. The 2‐year PFS was 96% and 10‐year PFS was 82% (Figure 1A). The 2‐year OS was 96% and 10‐year OS was 87% (Figure 1B). Cox regression was used to compare the survival probability between the following variables: Age, Sex, Spleen size, TLC and the scoring systems (Sokal, EUTOS and ELTS). The patients were divided into two groups based on: age (≤ 14 and > 14 years), spleen size (≤ 8 and > 8 cm) and TLC (≤ 1 lakh and > 1 lakh); and the survival compared. There was no significant difference observed in progression‐free survival between the age groups (< 14 and ≥ 14 years) and sex. On univariate analysis, spleen size, EUTOS scoring and CHR at 3 months were significantly associated with PFS. On multivariate analysis, only CHR at 3 months was predictive of PFS (*p* < 0.05) (Figure 2). EUTOS scoring system (*p* = 0.059) (Figure 3) and TLC > 1 L (*p* = 0.09) were also closely associated with PFS but did not reach statistical significance. Sokal scoring system, ELTS scoring system (Figure 4), EMR, MMR at 12 and 18 months were not predictive of PFS.

## 4 Discussion

The incidence of CML is rare in the paediatric population as compared to adults. The use of imatinib, a first‐generation TKI, has transformed the treatment paradigm of CML. Recently, the second line TKIs (dasatinib, nilotinib) have been approved for paediatric CML. In the present study, we have made an attempt to evaluate the effect of imatinib therapy on remission and long‐term outcome of paediatric CML CP patients.

Several past studies have reported that children and adolescents with CML tend to have a more aggressive clinical presentation than adults [3, 4, 5]. But it is debatable if this has any impact on the outcome. We found that the proportion of patients was slightly higher in the ≤ 14 years age group. This observation was different from the findings of previous Indian, Japanese and Asian groups where the majority of patients were more than 14 years [11, 12, 13]. Like earlier reports, a male preponderance was seen with a male: female ratio of 1.6:1 [13].

The present study reported abdominal fullness, asthenia, fever and weight loss as the major symptoms. These findings were parallel to past studies in Western and Indian settings who had demonstrated similar symptomatology [14, 15]. Splenomegaly was the most prominent physical sign, seen in 100% of our study population with a median spleen size of 10 cm below the costal margin. Previous Indian studies too had reported that 90%–100% of patients presented with splenomegaly [13, 14]. The published western literature quotes a lesser incidence of splenomegaly compared to Indian studies [15]. This could be due to frequent annual health checkups in developed nations, which pick up the disease in its indolent stage, before gross splenomegaly develops.

The median baseline Hb in the study population was 8.6 g/dL (range 4.4–17.8), median platelet count was 317 × 10^9^/L (range 84–1611) and median TLC was 217 000 cells/L (range 6–620). Our median TLC was slightly than the published literature showing median TLC of 170–180 × 10^9^/L. [13, 15] Thus, paediatric CML consistently has higher baseline TLC compared to the median TLC reported in various studies on adult CML [16, 17, 18]. In our study, the majority of the patients had e14a2 transcript (56.7%). This finding is again comparable to the various studies on paediatric and adolescent CML reporting 53%–68% patients having e14a2 transcript [13, 15]. We categorised the patients according to the validated risk stratification systems, namely Sokal, EUTOS and ELTS scoring systems. We found that 6.7%, 34.6% and 9.6% patients had high risk disease according to Sokal, EUTOS and ELTS scoring, respectively. The I CML Ped study reported Sokal high risk in as many as 55% patients [19]. However, other studies showed lower Sokal high‐risk patients between 25% and 35% [13, 20]. Similarly, EUTOS scoring also showed a wide variation among studies between 18% and 39% patients having high risk [13, 20]. Similar to our study, Froment et al. showed ELTS high risk in only 9% patients [15].

Imatinib was administered at an initial dose of 260 mg/m^2^. In children failing to achieve EMR or MMR at 12 months, the dose was increased to 340 mg/m^2^. 2nd generation TKIs became available to us only after 2016. Since this was a retrospective study, all included patients prior to 2016 who did not achieve response milestones received higher dose imatinib. Even after second‐generation TKIs became available, many patients could not receive these drugs due to resource constraints. These patients were also continued on higher dose imatinib at 340 mg/m^2^. We did switch a small subset of more recent imatinib failure patients to nilotinib or dasatinib, when feasible. Current NCCN and ELN guidelines recommend switching to second‐generation TKI in case of imatinib failure. Hence, our approach of dose escalation was more historical and not current best practice and might have also confounded overall response in CML.

Overall, 95.2% patients achieved CHR at 3 months and 54.8% patients achieved EMR at 3 months. A total of 42.5% and 54.4% patients achieved MMR at 12 and 18 months, respectively. Previous studies from low middle income countries (LMIC) also showed high CHR between 95.7% and 100% [20, 21]. I CML Ped study reported MMR at 12 months in 36% adolescent patients [15]. An Italian and a French study showed 66.6% and 31% MMR at 12 months, respectively [22, 23]. In Indian settings, a number of studies had evaluated outcomes in paediatric CML. A large study including 124 patients reported 50.9% MMR at 12 months [24]. Dave et al. and Kesana et al. showed 45% and 19% patients achieving MMR at 12 months [25, 26]. Madabhavi et al. found that 40.7% patients achieved MMR at 18 months whereas Kesana et al. found a much lower 18 month MMR of 27% [26, 27]. Thus, with the exception of Kesana et al., the majority of Indian studies showed consistent MMR hovering between 40% and 50%. Hence, our study had outcomes very similar to published literature.

The validity of the risk scores has not been established in paediatric settings. Sokal scoring was defined in the pre‐imatinib era with only a few children in the original study population [28]. EUTOS and ELTS scores were defined in an exclusively adult population [29, 30]. The French National Phase IV trial on paediatric CML by Millot et al. failed to find any correlation of Sokal score with cytogenetic response [23]. In Indian settings, Ganta et al. reported that Sokal low‐risk and EUTOS low‐risk patients had a higher chance of attaining complete cytogenetic response, but it was not statistically significant [31]. Vanik et al. and Kesana et al. also showed that Sokal and EUTOS scores were not significantly associated with molecular response [20, 26]. Hence, both western and Indian literature suggest that adult risk stratification scores are not predictive of response in paediatric CML. In our study, we found that Sokal score was significantly associated with MMR at 12 months but lost significance when correlated with MMR at 18 months. EUTOS and ELTS scores were not predictive of molecular response. Hence, our findings were a little different from published literature when it comes to Sokal scoring, but larger cohorts are required for validation.

In our study, EMR at 3 months was a significant variable in predicting MMR at 12 and 18 months. Our findings are validated by the French Glivec Phase IV study which also showed that patients achieving EMR at 3 months had higher rates of CCyR and MMR at 12 months [32]. Age group, spleen size, baseline TLC and transcript type were not predictive of molecular response in our study. There are a few studies which suggested that transcript e13a2 is associated with poorer response to therapy [33, 34].

In the present study, 2‐year PFS was 96% and 10‐year PFS was 82%. We found that CHR at 3 months was significantly predictive of PFS. We further found that patients with EUTOS high risk and those presenting with TLC > 100 × 10^9^/L at baseline had poorer PFS but these findings were not statistically significant. Sokal and ELTS scoring did not predict PFS. Hasford et al. showed significantly better 5‐year PFS of 90% in EUTOS low‐risk patients versus 82% in high‐risk patients [29]. On the other hand, Ganta et al. reported that Sokal and EUTOS scores were not predictive of EFS [31]. Furthermore, we did not find any correlation of EMR at 3 months and MMR at 12 months with PFS. This finding is in contrary to Millot et al. who showed that patients achieving EMR at 3 months had significantly better PFS at 36 and 48 months [32]. Similarly, Ganguly et al. derived that failure to achieve CCyR at 12 months predicted poor EFS [24]. Dave et al. also reported superior survival in patients achieving EMR at 3 months and MMR at 12 months [25]. Another Indian study achieved 8‐year EFS and OS of 43.1% and 80.4%, respectively. They also reported that MMR at 12 months was associated with better EFS [26]. The GIPAP cohort, consisting of various LMIC in Asia, Africa, Latin America and Eastern Europe, reported 3‐year OS of 89.4% [11]. In our study, 2‐ and 10‐year OS was 96% and 87%, respectively. Hence, we had excellent survival outcomes comparable to both Western and Indian literature.

A number of 11/104 (10.5%) patients progressed to blast crisis, with the majority of them (10/11) expiring due to disease. The one surviving patient has received intensive chemotherapy + TKI and is currently awaiting hematopoietic stem cell transplant (HSCT). Another Indian study had 2/73 patients (2.7%) progressing to blast crisis, both of whom succumbed. This finding again validates the very poor prognosis of CML BC.

Our study identified 25.7% patients who were non adherent to therapy. For children and adolescents, these numbers may be high. This could be attributed to affordability issues or a lack of interest to continue therapy after symptoms resolve and quality of life improves. Although 25.7% of patients had adherence issues, we observed excellent PFS and OS. Published studies show nonadherence is associated with worse event‐free survival and lower cytogenetic/molecular responses. Several factors may explain our findings: (1) our adherence assessment was retrospective and may under‐ or over‐estimate true pill‐taking (recall and documentation bias); (2) many nonadherence episodes were short interruptions rather than prolonged discontinuation and may not have lasted long enough to affect long‐term outcomes substantially; (3) a proportion of our cohort may have had favourable biology; and (4) close molecular monitoring may have detected non‐adherence much earlier than overt progression and helped in counselling and restarting therapy, which ultimately led to better survival.

In pre‐TKI era, allogenic HSCT was considered the only curative option in CML CP patients. With the advent of TKIs, HSCT is reserved for the very few patients with multiple TKI resistance or intolerance or patients with poor hematopoietic recovery with TKI [35]. On the other hand, HSCT remains the standard of care in CML BC. None of our patients underwent HSCT since the majority of them, fortunately, eventually responded to a higher dose of imatinib or a second‐ or third‐generation TKI. Due to the high‐financial burden of HSCT, a few patients who had not achieved MMR opted for continuation of TKI over HSCT. One patient of CML BC, post‐intensive chemotherapy + dasatinib, is currently awaiting allogenic HSCT.

A few studies reported the adverse effect of TKI on growth of children. A significant decrease in serum IGF 1 level has been found in children on TKI [36]. Further, a Chinese study reported that there was no difference in growth restriction between imatinib and dasatinib [37]. Since ours was a retrospective study, we could not monitor children for growth measurements at different time points. Prospective studies involving a larger study population are required to assess the growth velocity of children on TKI and compare to the control population.

## 5 Conclusion

This study presents one of the largest cohorts of paediatric CML CP patients in LMIC. In our study, 42.5% and 54.4% of patients achieved MMR at 12 and 18 months, respectively, which is comparable to outcomes in the Western population. We found that the Sokal score was significantly associated with MMR at 12 months but lost significance at 18 months. We also report excellent PFS and OS, with CHR at 3 months being the only predictor for PFS. None of the scoring systems could significantly predict survival outcomes. Thus, the traditional scoring systems for adults might not be fit, and the concept of ‘one size fits all’ cannot be applied. Identifying newer surrogate markers and scoring systems for early identification of high‐risk patients is of paramount importance.

## Author Contributions

**Souvik Saha:** conceptualization, writing – original draft, investigation, methodology, writing – review and editing, formal analysis, data curation. **Sanjeev yadav:** writing – review and editing, supervision. **Manish Kumar Singh:** writing – review and editing, supervision. **Khaliqur Rahman:** writing – review and editing, supervision. **Dinesh Chandra:** writing – review and editing, supervision. **Ruchi Gupta:** writing – review and editing, supervision. **Rajesh Kashyap:** conceptualization, writing – review and editing, visualization, validation, supervision.

## Ethics Statement

The study was approved by Institution Ethics Committee (IEC) vide 2025‐252‐DM‐EXP‐66. Waiver of consent was approved for the study by IEC in view of the retrospective study and data obtained from records with no direct patient contact.

## Conflicts of Interest

The authors declare no conflicts of interest.

## Data Availability

The data that support the findings of this study are available from the corresponding author upon reasonable request.
